# Circulating tumor DNA methylation marker MYO1-G for diagnosis and monitoring of colorectal cancer

**DOI:** 10.1186/s13148-021-01216-0

**Published:** 2021-12-27

**Authors:** Wu-Hao Lin, Jian Xiao, Zi-Yi Ye, Da-Liang Wei, Xiao-Hui Zhai, Rui-Hua Xu, Zhao-Lei Zeng, Hui-Yan Luo

**Affiliations:** 1 Department of Medical Oncology, Sun Yat-Sen University Cancer Center, State Key Laboratory of Oncology in South China, Collaborative Innovation Center for Cancer Medicine, Sun Yat-Sen University, 651 Dong Feng Road East, Guangzhou, 510060 People’s Republic of China; 2 Research Unit of Precision Diagnosis and Treatment for Gastrointestinal Cancer, Chinese Academy of Medical Sciences, Guangzhou, 510060 People’s Republic of China; 3 Faculty of Medical Sciences, Sun Yat-Sen University, Guangzhou, 510080 People’s Republic of China; 4 Department of Medical Oncology, The Sixth Affiliated Hospital of Sun-Yat Sen University, Guangzhou, 510655 People’s Republic of China

**Keywords:** Colorectal cancer_1_, CtDNA_2_, Methylation biomarker_3_, Diagnosis_4_, Monitoring_5_

## Abstract

**Background:**

Circulating tumor DNA (ctDNA) is a promising diagnostic and prognostic marker for many cancers and has been actively investigated in recent years. Previous studies have already demonstrated the potential use of ctDNA methylation markers in the diagnosis and prognostication of colorectal cancer (CRC). This retrospective study validated the value of methylation biomarker MYO1-G (cg10673833) in CRC diagnosis and disease monitoring using digital droplet PCR (ddPCR), a biomarker selected from our previous study due to its highest diagnostic efficiency.

**Methods:**

Blood samples of CRC and control samples from tumor-free individuals at two institutions were collected to quantify the methylation ratio using ddPCR. Area under curve (AUC) was calculated after constructing receiver operating characteristic curve (ROC) for CRC diagnosis. Sensitivity and specificity were estimated and comparisons of methylation ratio in different groups were performed.

**Results:**

We collected 673 blood samples from 272 patients diagnosed with stage I-IV CRC and 402 normal control samples. The methylation biomarker discriminated patients with CRC from normal controls with high accuracy (area under curve [AUC] = 0.94) and yielded a sensitivity of 84.3% and specificity of 94.5%. Besides, methylation ratio of MYO1-G was associated with tumor burden and treatment response. The methylation ratio was significantly lower in patients after their radical operation than when compared with those before surgeries (*P* < 0.001). Methylation ratio was significantly higher in patients with disease progression than those with stable disease (*P* = 0.002) and those with complete response or partial response (P = 0.009).

**Conclusions:**

Together, our study indicated that this methylation marker can serve as a potential biomarker for diagnosing and monitoring CRC.

**Supplementary Information:**

The online version contains supplementary material available at 10.1186/s13148-021-01216-0.

## Background

According to GLOBOCAN, colorectal cancer (CRC) accounted for 10% of global new cancer cases and has now become the fourth leading cause of cancer death worldwide [1]. Stage at diagnosis is the most important prognostic factor for cancer [2]. Earlier stages of CRC are associated with better survival of CRC patients [3]. Therefore, early detection has a significant effect on improving the prognosis of CRC patients. Colonoscopy is the most common and primary recommended method of CRC screening; however, it is not accepted by all people due to its cost and invasiveness. Carcinoembryonic antigen (CEA) is the only blood-based non-invasive method available in CRC surveillance but shows limited success due to its low sensitivity [4]. Furthermore, longitudinal monitoring of cancer recurrence after surgery and responses to therapy also plays a significant role in the management of CRC. Accordingly, this emphasizes the need to develop a novel, non-invasive, and more effective method for the early detection and monitoring of cancer recurrence and treatment response of CRC.

Circulating tumor DNA (ctDNA), carrying tumor-derived genetic alterations, has been actively investigated for its clinical application as a diagnostic biomarker and surrogate for monitoring treatment response in recent years [5, 6]. SEPT9 is one of the few validated methylation markers available in colorectal cancer [7–9]. Multiple retrospective studies have demonstrated that the sensitivity of SEPT9 ranged from 48 to 90% and the specificity ranged from 73 to 97% [9]. While prospective studies have found lower sensitivity (48–68%) and specificity (80–92%) in CRC [10]. Another non-invasive methylation biomarker that has frequently been described for CRC diagnosis is methylation of the VIM gene. VIM methylation showed a sensitivity and specificity of up to 59% and 93% in blood samples, respectively [11, 12]. A two-markers blood test for methylated BCAT1 and IKZF1 reached a sensitivity of 66% and a specificity of 94% for CRC detection in a prospective study, which enrolled 2107 individuals including 129 CRC patients [13].

However, few potential biomarkers are currently being used in monitoring treatment response in clinical practice. Our previous study has already demonstrated the usefulness of ctDNA methylation markers for diagnosis, prognostication, and surveillance of CRC and showed excellent diagnostic and prognostic prediction performance [14]. Our previous study first identified CRC-specific methylation signatures by comparing CRC tissues to normal blood leukocytes. Then, a machine-learning algorithm was applied to develop a predictive diagnostic (including 9 methylation markers) and a prognostic model (including 5 methylation markers) using cell-free DNA (cfDNA) samples from a cohort of 801 patients with CRC and 1021 normal controls. We found that the methylation status of CpG site cg10673833 demonstrated great efficiency both in diagnostic and prognostic capabilities in CRC.

Compared with panels with multiple biomarkers, a single biomarker testing kit is simpler and less expensive, which would promote universal testing in CRC patient or people at high risk of CRC. Droplet digital PCR (ddPCR), is one of the most commonly used dPCR, which is based on limiting partition of the PCR reaction volume and on poisson statistics to reduce the competition from any background DNA, allows precise detection of minimal amounts of a target of interest and reproducible data versus Real Time PCR [15–17]. Currently, the ddPCR system has actively been investigated for the detection of rare mutations, copy number variations, and gene rearrangements [18]. Nevertheless, only few studies applying ddPCR technology in DNA methylation analyses have been published so far [19]. Compared to NGS approaches, ddPCR experiments are easier and faster for a single known target. Furthermore, it requires smaller samples and present higher sensitivity up to 0.001% [20]. DdPCR technology does not require complex bioinformatics analysis. This study attempted to validate the potential use of methylated cg10673833 (MYO1-G) for CRC diagnosis and disease monitoring with a large sample size using ddPCR methods.

## Results

### Patient characteristics

We collected 673 blood samples from 272 patients diagnosed with stage I-IV CRC and 402 normal blood samples from the Sun Yat-sen University Cancer Center in Guangzhou, and the Sixth Affiliated Hospital of Sun Yat-sen University, China, from November 2019 until September 2020. All participants provided written informed consent for exploratory researches. Additional file 1: Table S1 and Additional file 2: Table S2 summarize the baseline characteristics of the patients and samples in the different groups. There were more female individuals in the group of normal controls who received routine physical examination in our hospital and with no results indicating disease (*P* = 0.009). CRC patients were older than the normal control group (*P* < 0.001). The median age of CRC patients and healthy controls was 56.5 (interquartile range [IQR] 50.0–65.0) and 45.0 (IQR 35.0–55.0) years old, respectively. There were 402 blood samples from CRC patients with tumor load and 271 blood samples from CRC patients without tumor load. CRC patients without tumor load referred to CRC patients who had already undergone treatment and no presence of disease could be detected by imaging examinations or colonoscopy. Among blood samples from CRC patients with tumor load that were used in diagnosis analysis, TNM stage I to IV accounted for 1.7%, 10.7%, 31.1%, and 56.5%, respectively.

### ctDNA methylation for diagnosis and molecular staging

**Fig. 1:**
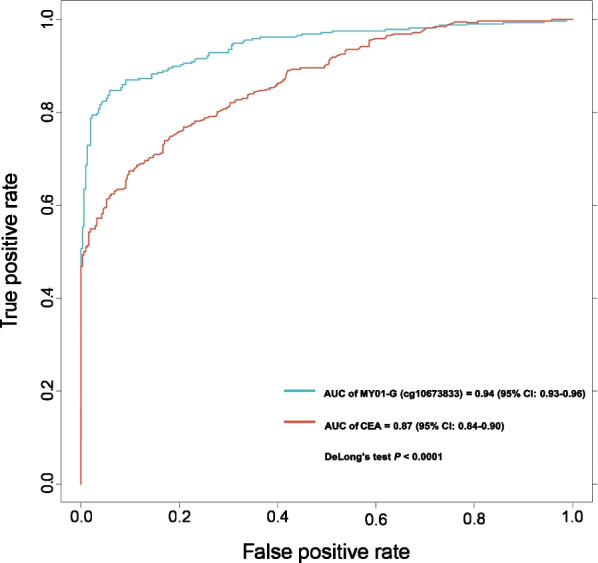
ROCs of MYO1-G methylation and CEA for CRC diagnosis. MYO1-G methylation value and CEA from 305 paired CRC samples and 307 normal controls were used in plotting the curve. The AUC of MYO1-G equaled to 0.94 and the AUC of CEA equaled to 0.87

**Fig. 2:**
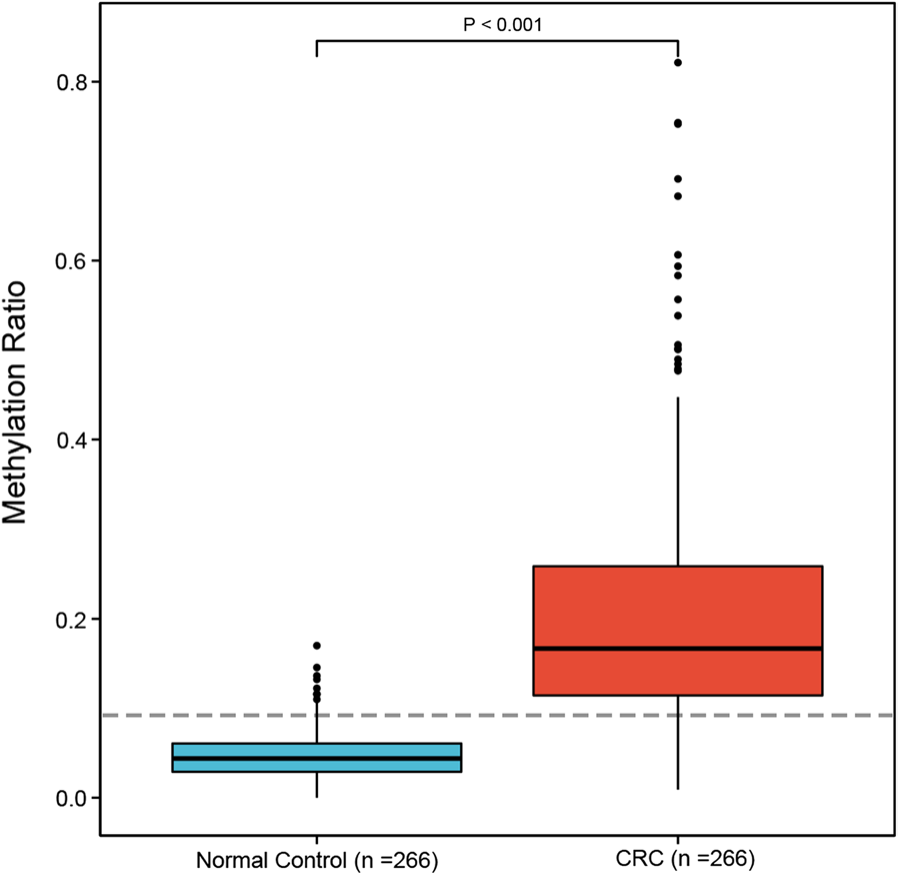
Boxplot of the methylation ratio in normal controls (*n* = 266) and CRC samples with tumor burden (*n* = 266) after propensity score matching

A total of 804 blood samples, including 402 normal controls and 402 blood samples from patients with detectable tumors, were enrolled in evaluating the potential usefulness of methylation of MYO1-G for CRC diagnosis. Samples with paired simultaneous CEA and ctDNA testing (*n* = 305 [CRC], n = 307 [normal controls]) were used to construct the ROCs and compare the AUCs. The ROC for CRC diagnosis is shown in Fig. 1, and the AUC of MYO1-G was 0.94 (95% CI 0.93–0.96), which indicated a good performance in distinguishing the CRC patients from healthy individuals. This biomarker performed significantly better than CEA (AUC of CEA = 0.87 [95% Cl 0.84–0.90], Fig. 1) (Delong’s test *P* < 0.0001). MYO1-G was hypermethylated in the ctDNA of CRC patients. Considering significant differences in age and gender between CRC samples and normal controls, propensity score matching was performed to yield a matched cohort to adjust these impacts when comparing the difference of the methylation ratio (Additional file 3: Table S3). The methylation ratio of CRC samples (n = 266) was significantly higher compared to normal controls (n = 266) (0.167 [IQR: 0.114—0.259] vs 0.044 [IQR: 0.029—0.061], P < 0.001, Fig. 2 and Additional file 1: Table S4). The cutoff value of MYO1-G methylation for CRC diagnosis in this study was set at 10% according to the results in our previous study [14]. For paired samples, the sensitivity of MYO1-G methylation for CRC diagnosis was significantly greater than CEA at a cutoff value of 5 µg/L (251 of 305 [82.3%] vs 167 of 305 [54.8%], P < 0.001). CEA showed a specificity of 97.1%. Table 1 shows the sensitivity and specificity of the paired ctDNA methylation test and CEA, respectively. For all CRC samples with tumor load, the methylation marker yielded a sensitivity of 84.3% and specificity of 94.5% when detecting CRC (Table 2). The positive predictive value (PPV) was 93.9% and the negative predictive value (NPV) was 85.8%.

**Table 1:** Sensitivity and specificity of the cfDNA paired ctDNA methylation test and CEA in patients with tumor load and normal controls

Biopsy finding	ctDNA methylation test (*n* = 612)*					CEA test (*n* = 612) *				
	*n*	Positive results (*n*)	Negative results (*n*)	Sensitivity %	Specificity %	*n*	Positive results (*n*)	Negative results (*n*)	Sensitivity %	Specificity %
Stage I	3	3	0	100.0		3	0	3	0.0	
Stage II	32	21	11	65.6		32	9	23	28.1	
Stage III	85	66	19	77.6		85	35	50	41.2	
Stage IV	185	161	24	87.0		185	123	62	66.5	
Total	305	251	54	82.3		305	167	138	54.8	
**Normal controls**	307	14	293		95.4	307	9	298		97.1

^*^Patients with tumor load were included in the sensitivity analysis

**Table 2:** Sensitivity and specificity of the ctDNA methylation test and CEA in patients with tumor load

Biopsy finding	ctDNA methylation test (*n* = 804)*				
	*n*	Positive results (*n*)	Negative results (*n*)	Sensitivity %	Specificity %
Stage I	7	6	1	85.7	
Stage II	43	32	11	74.4	
Stage III	125	104	21	83.2	
Stage IV	227	197	30	86.8	
Total	402	339	63	84.3	
Normal controls	402	22	380		94.5

**Fig. 3:**
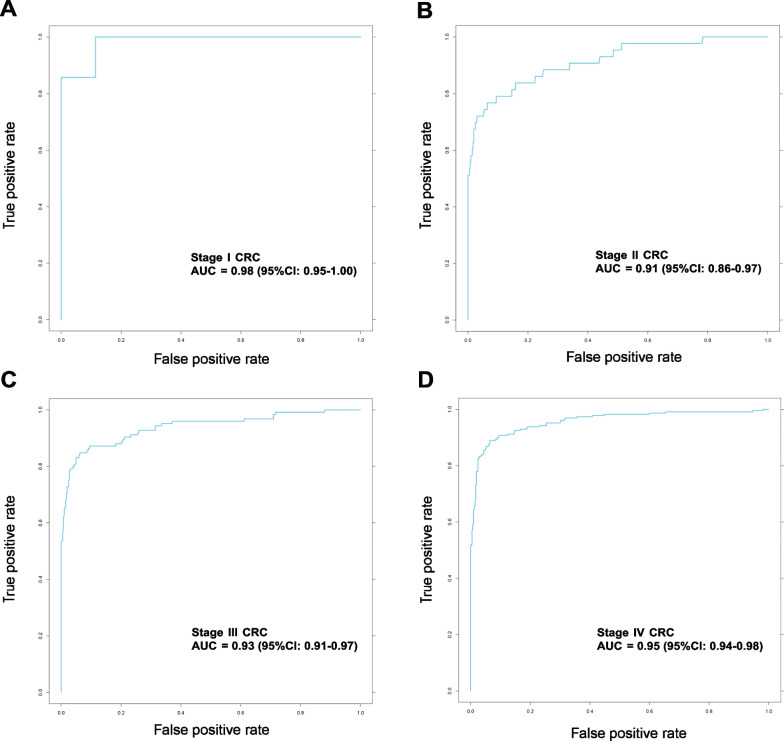
ROCs of MYO1-G methylation for stage I–IV CRC diagnosis, respectively. **A** Stage I CRC (*n* = 7), AUC = 0.98. **B** Stage II CRC (*n* = 43), AUC = 0.91. **C** Stage III CRC (*n* = 125), AUC = 0.93. **D** Stage IV CRC (*n* = 227), AUC = 0.95

**Fig. 4:**
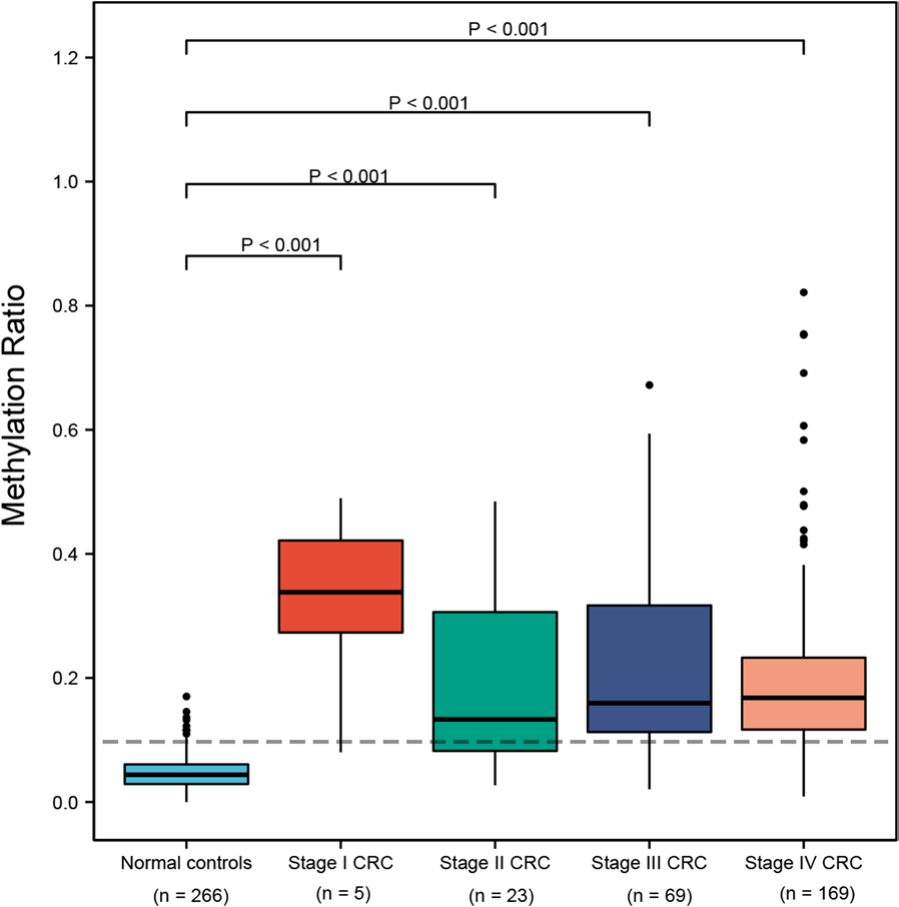
Boxplot of the methylation ratio in normal controls (*n* = 266) and stage I–IV CRC samples with tumor burden after propensity score matching (*n* = 5, 23, 69, 169 for stage I-IV CRC samples, respectively)

Taking disease stages into account, AUCs for Stage I–IV were 0.98 (95% CI 0.95–1.00), 0.91 (95% CI 0.86–0.97), 0.93 (95% CI 0.91–0.97) and 0.95 (95% CI 0.94–0.98), respectively (Fig. 3). The sensitivities for Stage I-IV were 85.7%, 74.4%, 83.2% and 86.8%, respectively (Table.2), and the sensitivity was not correlated with disease stages (*P* = 0.225, Pearson correlation). Stage I-IV samples showed a higher methylation ratio than normal controls (*P* < 0.001, Fig. 4). No significant differences among stage I-IV CRC (Fig. 4). Statistical description was listed in Additional file 4: Table S4.

### ctDNA methylation for disease monitoring

**Fig. 5:**
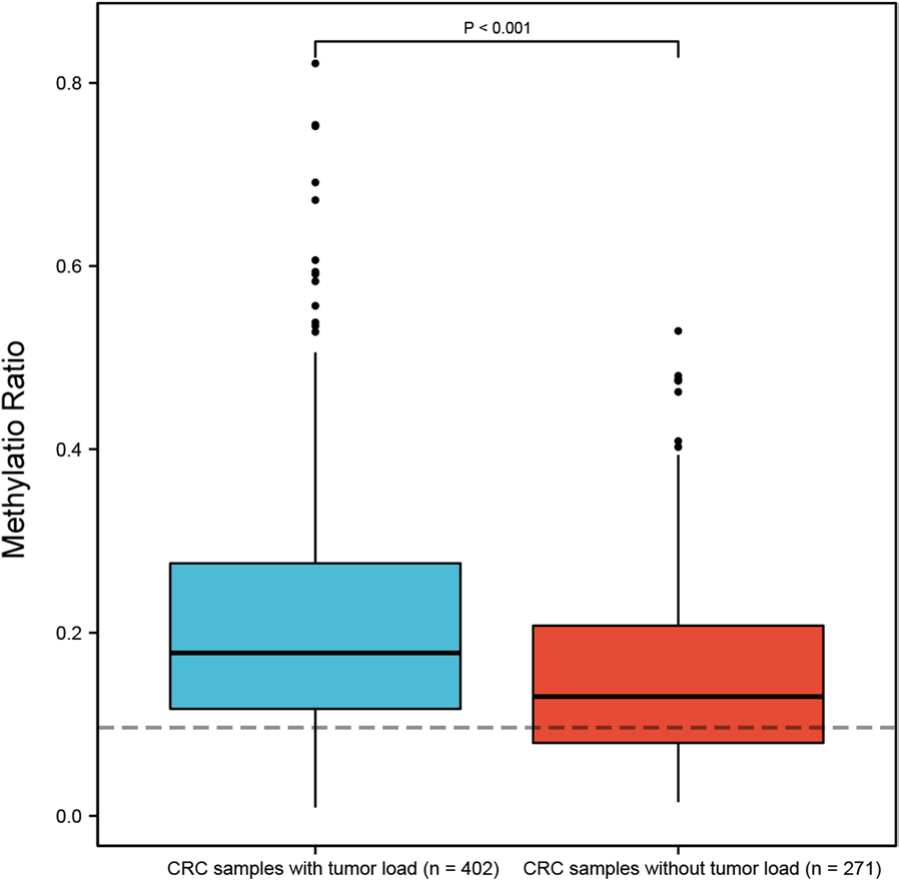
Boxplot of the methylation ratio in CRC samples with (*n* = 402) and without detectable tumor burden (*n* = 271)

**Fig. 6:**
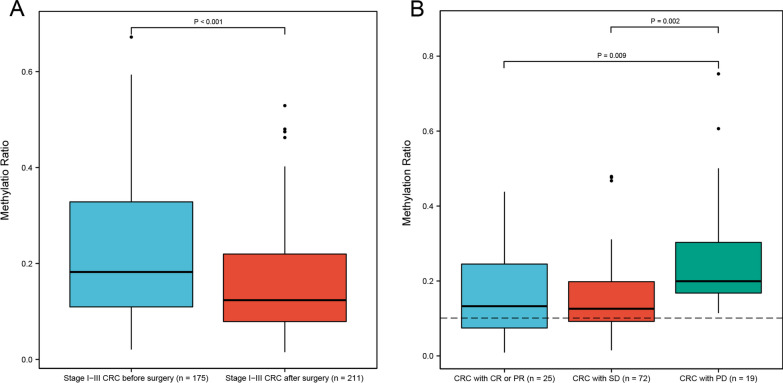
Methylation marker can serve as a potential biomarker for monitoring CRC. **A** Stage I–III CRC samples (not matched data) before surgery (n = 175) and after surgery (*n* = 211). **B** Methylation ratio in stage IV CRC samples (not matched data) with partial response (PR) or complete response (CR) (*n* = 25), stable disease (SD) (*n* = 72), and with progression (PD) (*n* = 19)

We next studied the utility of the methylation value in assessing surgery and treatment response of CRC. Methylation ratio in CRC patients with detectable residual tumor following treatment was significantly higher than those with no detectable tumor (0.18 [IQR: 0.12–0.28] vs. 0.13 [IQR: 0.08–0.21], *P* < 0.001, Fig. 5). The methylation ratio of MYO1-G was associated with tumor load (*P* < 0.001 OR [odds ratio] 60.8, 95% CI 14.6–275.1). Considering the presence of minimal residual tumor following surgery in some stage IV patients, we only enrolled stage I–III CRC patients for surgery assessment. A total of 386 CRC blood samples from 163 patients, including 175 pre-operation samples and 211 post-operation samples were analyzed. Among these samples, 58 were tested at only one time-point per patient and 328 samples were from the patients who were monitored over time. For patients with multiple time-points monitoring, the median follow-up was 2.5 months (IQR 1.2–5.2 months). Methylation ratios were significantly lower in patients after surgery than those before surgery (*P* < 0.001, Fig. 6A). In addition, the dynamic changes in ctDNA methylation were consistent with treatment outcomes. Among the stage IV samples for treatment response analysis, 32 were tested at one time-point only per patient and 84 samples were from the patients who were monitored over time. The median follow-up was 3.5 months (IQR 2.1–9.6 months). Those with a positive or stable treatment response (including complete response [CR], partial response [PR] and stable disease [SD]) had a significant concomitant decrease in ctDNA methylation compared to those with progressive disease [PD] (*P* = 0.009 [CR or PR versus PD], P = 0.002 [SD versus PD], Fig. 6B). Furthermore, at the cutoff value of 10%, 100% (19/19) PD samples tested positive, and the test positivity for SD and PR/CR were 69.4% (50/72) and 60.0% (15/25), respectively. Moreover, the methylation ratio of MYO1-G is associated with treatment response (*P* = 0.006, OR 84.7, 95% CI 3.8–2350.0).

**Fig. 7:**
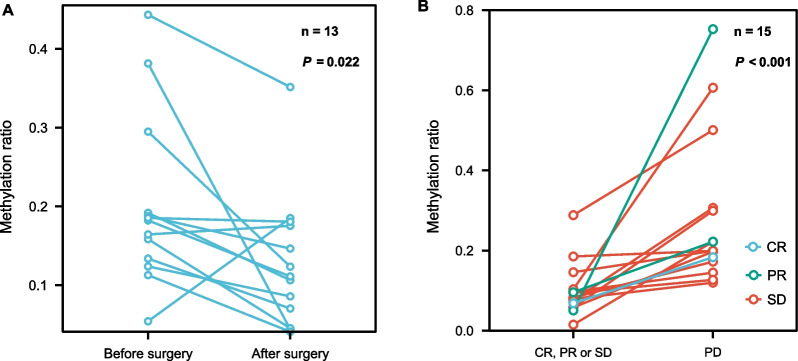
Methylation values correlated with treatment outcomes in CRC patients with matched plasma samples. **A** Changes in methylation ratio in stage I-III patients who had matched plasma samples before and after surgery (*n* = 13). **B** Changes in methylation ratio in stage IV patients who initially had complete response (CR), partial response (PR) or stable disease (SD) and then later had disease progression (PD) (*n* = 15)

Stage I-III CRC patients with both matched pre- and post-surgery data and samples collected within two months after surgery (*n* = 13) were utilized in paired data analysis. For individuals with matched pre- and post-surgery data, a significant decrease in methylation level was found in patients after surgery (*n* = 13, *P* = 0.022, Fig. 7A). Moreover, in patients with multiple time-point samples and changes in treatment response, patients with CR, PR or SD treatment responses showed a significantly lower methylation level than that of PD patients (*n* = 15, *P* < 0.001, Fig. 7B).

## Discussion

Our study highlighted the potential use of MYO1-G as an outstanding methylated biomarker for CRC diagnosis. The diagnostic accuracy (AUC) of our biomarker reached 0.94 with a sensitivity of 84.3% and specificity of 94.5%. It performed much better than CEA, especially in the early stages of the disease. Circulating methylated SEPT9 DNA was approved by the FDA in 2016 as the first molecular blood-based assay for CRC screening and was reported to have a sensitivity between 51 and 90% and specificity between 88% and 91.4% by previous studies [7, 8]. The sensitivity of our biomarker was comparable with SEPT9. It is noteworthy that the specificity in our study was up to 94.5%, which was higher than previous studies. A higher specificity would bring a higher positive predictive value when the test was put in clinical practice, which may avoid wasting medical resources especially in high-risk populations. We also demonstrated that CRC patients could be accurately differentiated from normal controls. Furthermore, we found that the methylation ratio of MYO1-G associated with tumor burden. Minimal residual disease (MRD) after surgery or therapy with curative intent is the main reason for cancer recurrence. Previous studies have reported that ctDNA methylation markers can prompt the presence of MRD, even in the absence of any other clinical evidence of disease, and can also identify patients with high risk of relapse. When taking tumor stages into consideration, even though the sample size of early stages CRC in our study was relatively small, stage I–II samples both showed a higher methylation level than normal controls, which indicated the effective early detection potential of CRC, while no significant differences in methylation levels were found among stage I–IV CRC. One possible explanation is that DNA methylation changes in tumor-related genes are frequent and early events during carcinogenesis, and transcriptional silencing of these genes by CpG methylation may continually contribute to the development of carcinomas [21, 22]. Considering the small sample size of stage I CRC and the lack of pre-cancerous samples, future studies should be conducted to confirm whether methylation of MYO1-G is hypermethylated in stage I disease and pre-cancerous tissue. As demonstrated in Table 1, although the sensitivity increased slightly as the stage advanced (except for stage I samples due to the small sample size), there was no significant correlation between sensitivity and disease stages. This test performed much better in patients with advanced stage (III–IV) CRC, of which the sensitivity of both stages was higher than 80%.

We next investigated the utility of the methylation value in the monitoring of CRC. The analysis of all patients who received surgery, including unmatched and matched patient samples, showed that the methylation levels significantly dropped after tumor removal. However, we also observed a small portion of patients with positive post-operation methylation levels; whether these positive results are associated with recurrence remains to be confirmed by further follow-up. In patients with disease recurrence, the methylation levels were higher than those with a tumor-free status. These results suggested that methylation of MYO1-G may be capable of identifying patients with a high risk of recurrence. Currently, treatment responses of CRC are evaluated by imaging with the Response Evaluation Criteria in Solid Tumors (RECIST). However, imaging evaluation has shortcomings such as relatively higher cost and the reliance on the radiologist’s experience. The association between biomarkers and treatment response has rarely been studied. Our study demonstrated that patients with stage IV CRC without disease progression had a lower methylation level than that of PD, which provided the potential for screening out patients who were resistant to current treatment, whereby further adjustments in treatment plan should be advised to patients whose methylation levels remained unchanged or increased.

There are several limitations to our study. First, this study was a retrospective study. Secondly, few cases with recurrence were found in our follow-up, which limited the analysis of change of methylation level after disease recurrence. Another limitation is that the median follow-up time for the patients with multiple monitoring was 2.5 month and the percentage of stage II–III CRC patients with a 3-year disease free survival was above 70% [23], such a follow-up period was not long enough to compare the recurrence rate for the post-operative patients with methylation value higher that 10% and those below 10%. Finally, the sample sizes of the early-stage CRC were relatively small. Collectively, these results suggest that the methylation level correlates well with tumor burden and may have utility in surveillance for recurrence and evaluation of treatment efficacy.

## Conclusions

In conclusion, the results of our study indicated that the methylation marker could serve as a potential biomarker for the diagnosis and monitoring of CRC, with also the potential to be used for the early detection of CRC and provide evidence of recurrence and cause for adjustment of treatment strategy.

## Methods

### Study design

The aim of this study was to validate the value of a ctDNA methylation–based biomarker MYO1-G for the diagnosis and monitoring of CRC. First, we calculated the sensitivity and specificity of our methylation biomarker and CEA. The clinical performance of MYO1-G was evaluated as a diagnostic test by comparison to CEA through receiver operating characteristic (ROC) curves and the associated areas under curves (AUCs). We then compared the difference between normal individuals and stage I-IV CRC patients. Differences of the methylation ratio among different stages were also analyzed. The TNM staging classification for CRC is according to the 8th edition of the AJCC cancer staging manual [24]. Further, to evaluate the monitoring potential of the methylation biomarker, we compared the methylation ratio in Stage I-III CRC patients before and after surgery, Stage IV CRC patients of different treatment responses according to RECIST criteria 1.1. Besides, the matched pre- and post- surgery data and paired different treatment response data from stage IV CRC patients were analyzed to confirm the results. In our study, “tumor burden” and “tumor load” refer to the detectable tumor found in CT or colonoscopy. Recurrence status of the patients was assessed by CT or colonoscopy.

### Patients and sample collection

272 patients with CRC from stage I–IV and healthy individuals were included from the Sun Yat-sen University Cancer Center in Guangzhou, and the Sixth Affiliated Hospital of Sun Yat-sen University, China. The diagnosis of CRC was confirmed by biopsy. The healthy controls were the population receiving routine physical examination in our hospital and those with no results indicating disease reported. Routine physical examination included blood test, blood biochemistry, common tumor biomarkers, B-ultrasonography of liver, gallbladder, spleen, pancreas and urinary system, chest radiography and colonoscopy. All participants provided written informed consent for exploratory researches. The median follow-up time for the patients with multiple monitoring after surgery  and during treatment response evaluation was 2.5 months and 3.5 months, respectively. And the interquartile range of follow-up time was 1.2–5.2 months and 2.1–9.6 months, respectively. Patients’ characteristics and tumor features are summarized in Table S1. 10 ml human blood samples were obtained as clinically indicated for patient care and were retained for this study and collected in EDTAK2 anticoagulation tubes by venipuncture. The blood samples were centrifuged at 1600*g* for 10 min to separate the plasma, then the plasma samples were centrifuged at 10,000*g* for 10 min, then the supernatant plasma was drawn and transferred to a 2.0 ml Eppendorf tube. Plasma samples were frozen and preserved at -80℃ until use. Samples that do not meet the testing requirements are excluded from analysis.

### Isolation and methylation profiling of cfDNA

Minimal 1.5 ml plasma samples were used to extract cfDNA using HiPure Circulating DNA Midi Kit (Magen, China) according to the manufacturer’s recommendations in our study. 10–20 ng of DNA was converted to bis-DNA using EpiTect Fast DNA Bisulfite Kit (QIAGEN) according to the manufacturer’s protocol. Resulting bis-DNA had a size distribution of ~ 200–3000 bp, with a peak around ~ 500–1000 bp. The efficiency of bisulfite conversion was > 99.8% as verified by deep-sequencing of bis-DNA and analyzing the ratio of C to T conversion of CH (non-CG) dinucleotides [25–27]. To measure the methylation status of MYO1-G, we adopted a droplet digital polymerase chain reaction (PCR) paradigm featuring a Bio-Rad QX-200 Droplet Reader and an Automated Droplet Generator (AutoDG). The primers and dual labeled fluorescent probes were custom designed and synthesized by Thermo Fisher Scientific [14]. The primer and probe sequences were as followed. Primer: cg10673833-F: 5'-GTTTTATAAGGAGGTTGTGTT-3'; cg10673833-R: 5'-AACIAAAAACCCTCCAAA-3'; Probe: cg10673833-M: 5'- FAM/GAGGGGTCGGATGTTGG/BHQ1-3'; cg10673833-NM: 5'-HEX/GAGGGGTTGGATGTTGGG/BHQ1-3'. The following cycling conditions were used: 98 °C for 10 min, followed by 40 cycles at 98 °C for 30 s and 53 °C for 60 s, and finally 98 °C for 10 min. The ddPCR™ Supermix for probes and other universal digital PCR reagents and protocols were purchased from Bio-Rad. The number of droplets was determined using a QX-200 droplet reader and analyzed using QuantiSoft software (Bio-Rad). Reactions were excluded if fewer than 10,000 droplets were counted. We used 10% methylation ratio as a cutoff according to the results in the previous study and no internal reference gene was needed in ddPCR, and a methylation ratio < 10% was defined as negative [14]. Methylation ratio = (methylated copies/[methylated copies + non-methylated copies] × 100).

### Statistical analysis

Diagnostic accuracy was described by using the means of ROC analysis. Areas under the curve (AUCs) were reported including 95% CIs. Normal distribution of methylation value in each clinical category was tested by Shapiro–Wilk normality test (*α* = 0.05). Propensity score matching was performed to yield a matched cohort to adjust the imbalanced factors. Wilcoxon test (for matched analysis), Mann–Whitney *U* test and Kruskal–Wallis test were adopted to compare the methylation level in different clinical categories due to the non-normal distribution of the data. Statistical significance was set as *P* < 0.05 in a two-tailed test. Statistical analysis was performed using R 3.5.1 (http://www.r-project.org).

### Supplementary Information

**Additional file 1: Table S1**. Patient Characteristics of the study cohort.

**Additional file 2: Table S2**. Sample characteristics of the study cohort.

**Additional file 3: Table S3**. Sample characteristics of the study cohort after propensity score matching.

**Additional file 4: Table S4**. Statistical description of the methylation ratio in different groups after propensity score matching.

**Additional file 5: Table S5**. Statistical description of the methylation ratio in different groups in disease monitoring analysis.

## Data Availability

The datasets used and/or analyzed during the current study are available from the corresponding author on reasonable request.
