# Supplementary material for: Circulating tumor DNA methylation marker MYO1-G for diagnosis and monitoring of colorectal cancer
Source: Clin Epigenetics. 2021 Dec 27;13:232. doi: 10.1186/s13148-021-01216-0 (PMC8713401; doi:10.1186/s13148-021-01216-0)
Supplement: Supplementary file 1 — Additional file 1: Table S1. Patient Characteristics of the study cohort. [file 13148_2021_1216_MOESM1_ESM.docx]

**Table S1 Patient Characteristics of the study cohort.**

|  | Normal controls | CRC patients | p.value |
| --- | --- | --- | --- |
| **Number** | 402 | 272 |  |
| **Gender = female (%)** | 212 (52.9) | 115 (42.3) | 0.009 |
| **Age, years**  **(median [IQR])** | 45.00 [35.00, 55.00] | 56.50 [50.00, 65.00] | <0.001 |
| **Stage (%)** |  |  | NA |
| I | NA | 11 (4.0) |  |
| II | NA | 43 (15.8) |  |
| III | NA | 107 (39.3) |  |
| IV | NA | 111 (40.8) |  |

NA, not applicable
