# Supplementary material for: Circulating tumor DNA methylation marker MYO1-G for diagnosis and monitoring of colorectal cancer
Source: Clin Epigenetics. 2021 Dec 27;13:232. doi: 10.1186/s13148-021-01216-0 (PMC8713401; doi:10.1186/s13148-021-01216-0)
Supplement: Supplementary file 2 — Additional file 2: Table S2. Sample characteristics of the study cohort. [file 13148_2021_1216_MOESM2_ESM.docx]

**Table S2 Sample characteristics of the study cohort.**

|  | **Normal blood samples** | **CRC blood samples from patients without tumor load** | **CRC blood samples from patients with tumor load** | **p.value** |
| --- | --- | --- | --- | --- |
|  |  |  |  |  |
| **Number** | 402 | 271 | 402 |  |
| **Gender = female (%)** | 213 (53.0) | 123 (45.4) | 172 (44.3) | 0.031 |
| **Age, years**  **(median [IQR])** | 45.00 [35.00, 55.00] | 56.00 [51.00, 65.00] | 57.00 [48.00, 64.75] | <0.001 |
| **CEA value** |  |  |  | NA |
| ＞5µg/l | 9 (2.2%) | 60 (22.1%) | 167 (41.6%) |  |
| ＜5µg/l | 298 (74.1%) | 170 (62.7%) | 138 (34.2%) |  |
| NA | 85 (23.6%) | 41 (15.1%) | 97 (24.2%) |  |
| **Stage** |  |  |  | NA |
| I | NA | 18 (6.6) | 7 (1.7) |  |
| II | NA | 53 (19.6) | 43 (10.7) |  |
| III | NA | 140 (51.7) | 125 (31.1) |  |
| IV | NA | 60 (22.1) | 227 (56.5) |  |

NA, not applicable
