# Supplementary material for: Circulating tumor DNA methylation marker MYO1-G for diagnosis and monitoring of colorectal cancer
Source: Clin Epigenetics. 2021 Dec 27;13:232. doi: 10.1186/s13148-021-01216-0 (PMC8713401; doi:10.1186/s13148-021-01216-0)
Supplement: Supplementary file 3 — Additional file 3: Table S3. Sample characteristics of the study cohort after propensity score matching. [file 13148_2021_1216_MOESM3_ESM.docx]

**Table S3 Sample characteristics of the study cohort after propensity score matching.**

|  | **Normal blood samples** | **CRC blood samples from patients with tumor load** | **p.value** |
| --- | --- | --- | --- |
|  |  |  |  |
| **Number** | 266 | 266 |  |
| **Gender = female (%)** | 136 (51.1) | 134 (50.4) | 0.931 |
| **Age, years**  **(median [IQR])** | 50.00 [44.00, 60.00] | 51.00 [43.00, 59.00] | 0.823 |
| **Stage** |  |  | NA |
| I | NA | 5 (1.9) |  |
| II | NA | 23 (8.6) |  |
| III | NA | 69 (25.9) |  |
| IV | NA | 169 (63.5) |  |

NA, not applicable
