# Supplementary material for: Circulating tumor DNA methylation marker MYO1-G for diagnosis and monitoring of colorectal cancer
Source: Clin Epigenetics. 2021 Dec 27;13:232. doi: 10.1186/s13148-021-01216-0 (PMC8713401; doi:10.1186/s13148-021-01216-0)
Supplement: Supplementary file 4 — Additional file 4: Table S4. Statistical description of the methylation ratio in different groups after propensity score matching. [file 13148_2021_1216_MOESM4_ESM.docx]

**Table S4. Statistical description of the methylation ratio in different groups after propensity score matching.**

| Group | n | Min | Max | Median | IQR | Lower quartile | Upper quartile | Mean | SD | SE |
| --- | --- | --- | --- | --- | --- | --- | --- | --- | --- | --- |
| Normal controls | 266 | 0 | 0.17 | 0.044 | 0.032 | 0.029 | 0.061 | 0.048 | 0.027 | 0.002 |
| CRC | 266 | 0.009 | 0.821 | 0.167 | 0.144 | 0.114 | 0.259 | 0.208 | 0.141 | 0.009 |
| Stage I CRC | 5 | 0.08 | 0.49 | 0.338 | 0.148 | 0.273 | 0.422 | 0.321 | 0.157 | 0.07 |
| Stage II CRC | 23 | 0.027 | 0.484 | 0.133 | 0.224 | 0.082 | 0.306 | 0.2 | 0.145 | 0.03 |
| Stage III CRC | 69 | 0.021 | 0.672 | 0.159 | 0.204 | 0.113 | 0.317 | 0.224 | 0.149 | 0.018 |
| Stage IV CRC | 169 | 0.009 | 0.821 | 0.168 | 0.116 | 0.117 | 0.233 | 0.199 | 0.136 | 0.01 |
