# Supplementary material for: Circulating tumor DNA methylation marker MYO1-G for diagnosis and monitoring of colorectal cancer
Source: Clin Epigenetics. 2021 Dec 27;13:232. doi: 10.1186/s13148-021-01216-0 (PMC8713401; doi:10.1186/s13148-021-01216-0)
Supplement: Supplementary file 5 — Additional file 5: Table S5. Statistical description of the methylation ratio in different groups in disease monitoring analysis. [file 13148_2021_1216_MOESM5_ESM.docx]

**Table S5. Statistical description of the methylation ratio in different groups in disease monitoring analysis.**

| Group | n | Min | Max | Median | IQR | Lower quartile | Upper quartile | Mean | SD | SE |
| --- | --- | --- | --- | --- | --- | --- | --- | --- | --- | --- |
| Stage I-III CRC before surgery | 175 | 0.021 | 0.672 | 0.182 | 0.219 | 0.109 | 0.328 | 0.225 | 0.146 | 0.011 |
| Stage I-III CRC after surgery | 211 | 0.015 | 0.529 | 0.124 | 0.141 | 0.079 | 0.22 | 0.156 | 0.104 | 0.007 |
| Stage IV CRC with CR or PR | 25 | 0.009 | 0.438 | 0.133 | 0.171 | 0.074 | 0.245 | 0.16 | 0.113 | 0.023 |
| Stage IV CRC with SD | 72 | 0.015 | 0.479 | 0.126 | 0.106 | 0.092 | 0.198 | 0.155 | 0.098 | 0.012 |
| Stage IV CRC with PD | 19 | 0.114 | 0.753 | 0.199 | 0.135 | 0.168 | 0.303 | 0.272 | 0.173 | 0.04 |
